# An Underwater Source Localization Method Using Bearing Measurements

**DOI:** 10.3390/s24051627

**Published:** 2024-03-01

**Authors:** Peijuan Li, Yiting Liu, Tingwu Yan, Shutao Yang, Rui Li

**Affiliations:** 1 Industrial Center, Nanjing Institute of Technology, Nanjing 211167, China; mach_ytw@163.com (T.Y.); 18252812244@163.com (S.Y.); 15195977181@163.com (R.L.); 2 School of Automation, Nanjing Institute of Technology, Nanjing 210096, China; gcdlyt1985@163.com

**Keywords:** USBL, bias compensation, SDP, bearing measurements, source localization, CRLB

## Abstract

Angle-of-arrival (AOA) measurements are often used in underwater acoustical localization. Different from the traditional AOA model based on azimuth and elevation measurements, the AOA model studied in this paper uses bearing measurements. It is also often used in the Ultra-Short Baseline system (USBL). However, traditional acoustical localization needs additional range information. If the range information is unavailable, the closed-form solution is difficult to obtain only with bearing measurements. Thus, a localization closed-form solution using only bearing measurements is explored in this article. A pseudo-linear measurement model between the source position and the bearing measurements is derived, and considering the nonlinear relationship of the parameters, a weighted least-squares optimization equation based on multiple constraints is established. Different from the traditional two-step least-squares method, the semidefinite programming (SDP) method is designed to obtain the initial solution, and then a bias compensation method is proposed to further minimize localization errors based on the SDP result. Numerical simulations show that the performance of the proposed method can achieve Cramer–Rao lower bound (CRLB) accuracy. The field test also proves that the proposed method can locate the source position without range measurements and obtain the highest positioning accuracy.

## 1. Introduction

Source localization is essential for many applications, such as radar [1], sensor networks [2], and underwater navigation [3]. It is inevitable to encounter synchronization problems with heterogeneous data clocks when different sensors are converged. Fortunately, localization using angle-of-arrival (AOA) measurements can effectively alleviate the problem of different clock periods, so it has attracted wide attention.

Traditional source localization methods include time-of-arrival (TOA), time-difference-of-arrival (TDOA), and AOA. Both TDOA and AOA have the advantage of not needing synchronized sensor clocks, and they are suitable for single-source passive navigation [3] or acoustical localization of a black box [4]. Traditional two-stage weighted least squares (TWLSs) are commonly used for TDOA-based localization problems due to their low computational complexity [5]. Several improved algorithms have been proposed, such as algorithms considering sensor error [6] or bias reduction [7]. Hybrid systems, such as TDOA/AOA [8] and TDOA/FDOA [9], can improve positioning accuracy. Thus, they have drawn considerable attention in recent years.

In traditional TDOA/AOA- or AOA-based localization problems, azimuth and elevation measurements are required to locate the source. Take the AOA localization model as an example. The closed-form solution is more attractive, thanks to its low computational complexity [10,11]. The weighted least-squares method is the most popular approach to the source localization problem. A Gauss–Newton maximum likelihood estimation (MLE) method has also been designed for AOA localization [12]. Several different AOA-based localization models have been researched. A bearings-only target localization model was proposed based on a total least-squares method in 2005 [13]. The localization model was analyzed in 2-D space. However, additional problems appeared, and the localization model would be different in the 3-D space. Therefore, a 3-D localization technique using 1-D AOAs of the source was proposed [14]. The directions of the linear arrays are needed for the model. 

Most research is focused on AOA models based on azimuth and elevation measurements [15,16]. In [17], a semidefinite programming (SDP) method was used to solve the problem of AOA localization, taking into account the unique characteristics of the device, such as the USBL, where only the bearings can be obtained. The localization model is different, and the traditional closed-form solution is not applicable. Thus, the source localization problem based on bearing angles is addressed in this paper. The source localization problem in underwater acoustical localization is greatly solved by utilizing bearing measurement technology. Moreover, researchers tend to use bearing measurements to solve acoustical localization problems [18,19]. According to the localization model described in several papers [3,18], bearing measurements can be applied in 3-D source localization problems when additional time-of-arrival (TOA) or time-difference-of-arrival (TDOA) measurements are available. Otherwise, if range measurements are unavailable, it is difficult to locate the source position. This is also the difficulty of applying the bearings localization model in 3-D space. The existing literature [18,20] has focused on the AOA-based localization problem with a traditional closed-form solution that is not suitable for this paper. One of the contributions of this paper is to derive a new closed-form solution based on bearing measurements.

Inspired by [3,18], where TDOA/AOA or TOA/AOA measurements are used for locating an underwater source position, this paper analyzes the AOA localization method. In complex underwater environments, TOA or TDOA measurements are insufficient for positioning in the presence of outliers. Thus, the AOA-based localization problem should draw considerable attention. The challenge of AOA-based localization is that a closed-form solution is difficult to obtain without range measurements. The core aim of this paper is to solve the problem of bearing-based AOA localization without range information.

The contributions of the paper are twofold:

(1) A new underwater AOA localization model is established, in which only bearing measurements are used. Considering the nonlinear relationship of the parameters, a weighted least-squares optimization equation based on multiple constraints is established. A semidefinite programming (SDP) method is designed to obtain the initial solution.

(2) A Taylor expansion strategy-based bias compensation method is developed to improve estimation accuracy, as noise in bearing measurements will affect positioning accuracy.

The structure of this paper is as follows: Section 1 is the introduction and includes the current research status, Section 2 introduces the system model, Section 3 introduces the localization method and analyzes the Cramer–Rao lower bound of the bearing-based AOA localization problem, Section 4 verifies the effectiveness of the proposed algorithm through simulation and field tests, and Section 5 presents a summary. 

## 2. System Model

**Figure 1:**
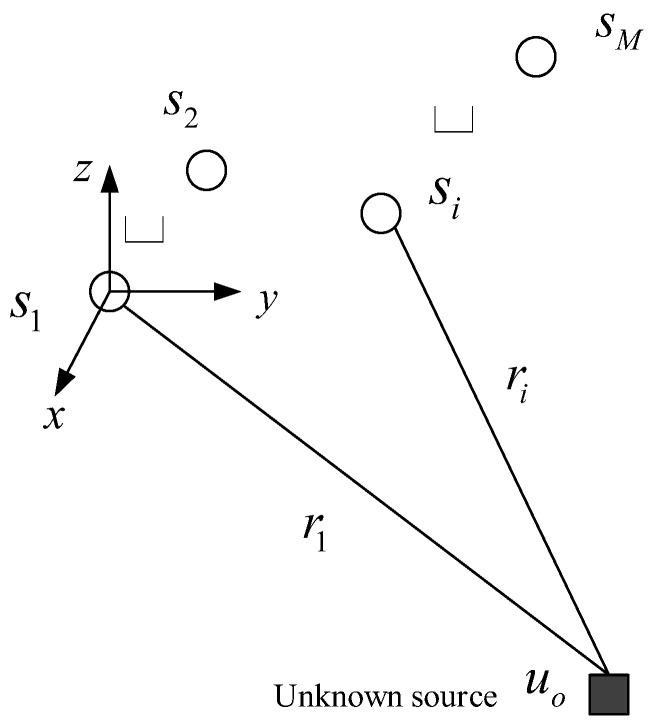
Source location diagram.

We assume that the true positions of the basic sensors, $s_{i}={\left(\begin{array}{ccc} x_{i} & y_{i} & z_{i} \end{array}\right)}^{T}\left(i=1,2,3,\ldots M\right)$, are known in advance, where M represents the number of basic sensors. The source position, $\left(u_{o}={\left(\begin{array}{ccc} x & y & z \end{array}\right)}^{T}\right)$, is unknown and needs to be located. Note that the sensors and the source are static, as Figure 1 shows.

The symbol $r_{i}$ denotes the slant distance between the source and sensor ($s_{i}$). In the context of the underwater Ultra-Short Baseline (USBL) system localization model as detailed in [20], considering the sensor $s_{i}$ specifically, the relationship between the bearings and the source’s position is subsequently illustrated.

**Figure 2:**
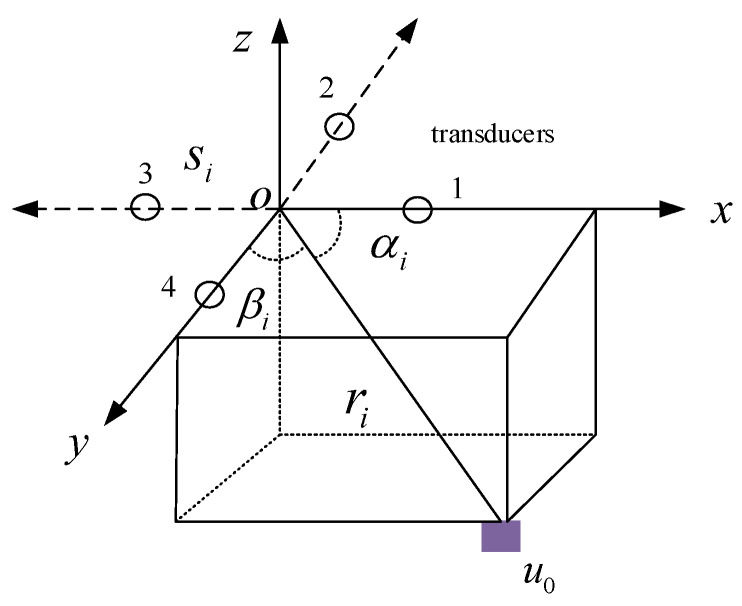
Bearing information diagram.

Usually, we assume that there are four transducers in each $s_{i}$, as shown in Figure 2, which are installed along the x- and y-axes. $\alpha_{i}$ and $\beta_{i}$ are local elevation angles relative to the x- and y-axes, where $\alpha_{i}$ is obtained from the transducer array along the x-axis and $\beta_{i}$ is obtained from the transducer array along the y-axis. These angles are ascertained based on the phase difference between the respective pairs of transducers. The phase difference is estimated by the adaptive phase difference estimator based on the Least Mean Square method [21]. The two angles can be used for localization. There are M pairs of angles $\left(\alpha ,\beta \right)$ can be obtained in the condition of $s_{1},s_{2},\ldots ,s_{M}$. In the traditional localization model, they need a slant distance to complete positioning. The localization model is as follows [3,22].

(1) $$\left\{\begin{array}{c} x-x_{i}=r_{i}cos\left(\alpha_{i}^{0}\right)\\ y-y_{i}=r_{i}cos\left(\beta_{i}^{0}\right) \end{array}\right.$$

where $\alpha_{i}^{0}$ and $\beta_{i}^{0}$ are the true value. $\left(x,y\right)$ is the horizontal coordinate of the source and $\left(x_{i},y_{i}\right)$ is the horizontal coordinate of the *i*th basic sensor. If the position of the *i*th basic sensor is known, the source position can be obtained then. Note that the x–y–z-axis in Figure 2 is the body frame. The source position is required to be established within the world coordinate frame. This issue can be resolved through the utilization of measured attitude data, which illustrates the dynamic relationship between the body coordinate frame and the world frame. Given known attitude information, it becomes feasible to reconcile the global and body coordinate frames into a unified framework. Considering that measuring the attitude falls outside the scope of this paper, it is presumed that the attitude information is known for the purposes of our discussion.

However, in the absence of a slant distance, only the angles are needed to complete the positioning. To describe the relationship between angle measurements and position, it can be represented as follows:

(2) $$\left\{\begin{array}{c} \alpha_{i}=arccos\left(\frac{x-x_{i}}{r_{i}}\right)+\varepsilon_{i}=\alpha_{i}^{0}+\varepsilon_{i}\\ \beta_{i}=arccos\left(\frac{y-y_{i}}{r_{i}}\right)+\delta_{i}=\beta_{i}^{0}+\delta_{i} \end{array}\right.$$

where $\varepsilon_{i}$ and $\delta_{i}\;$represent the measurement noise of bearing $\alpha_{i}$ and $\beta_{i}$. It should be noted that the noise vector $\varepsilon ={\left[\begin{array}{ccc} \varepsilon_{1} & \cdots & \varepsilon_{i} \end{array}\right]}^{T}$ and $\delta ={\left[\begin{array}{ccc} \delta_{1} & \cdots & \delta_{i} \end{array}\right]}^{T}$ are assumed to follow a zero-mean Gaussian distribution, i.e., $\varepsilon \sim N\left(0,\;Q_{\alpha }\right)$ and $\delta \sim N\left(0,\;Q_{\beta }\right)$, where $Q_{\alpha }=E\left(\varepsilon \varepsilon^{T}\right)$ and $Q_{\beta }=E\left(\delta \delta^{T}\right)$.

The localization model in 3-D space is different from that in paper [13] in 2-D space as the additional slant distance is needed in the 3-D space model. However, the slant distance is unknown and it is difficult to obtain a closed-form solution. 

Given the measurements $\alpha ={\left[\begin{array}{ccc} \alpha_{1} & \cdots & \alpha_{i} \end{array}\right]}^{T}$ and$\;\beta ={\left[\begin{array}{ccc} \beta_{1} & \cdots & \beta_{i} \end{array}\right]}^{T}$, our goal is to locate the source position in the case of unknown slant distance.

## 3. Localization Method

In this section, we propose an effective solution to the positioning model presented in the previous section.

### 3.1. Initial Solution

Firstly, the pseudo-linear equations are derived for the bearing model as follows. Based on the noisy values in (1), we can obtain

(3) $$\left\{\begin{array}{c} x-x_{i}-r_{i}cos\left(\alpha_{i}\right)=\eta_{\alpha }\\ y-y_{i}-r_{i}cos\left(\beta_{i}\right)=\eta_{\beta } \end{array}\right.$$

The small measurement noise $\varepsilon_{i}$ can lead to the results $cos\left(\varepsilon_{i}\right)\approx 1$ and $sin\left(\varepsilon_{i}\right)\approx \varepsilon_{i}$. Then, the following approximation can be obtained:

(4) $$cos\left(\alpha_{i}^{0}+\varepsilon_{i}\right)=\cos\alpha_{i}^{0}-\varepsilon_{i}sin\alpha_{i}^{0}$$

Take $x-x_{i}-r_{i}cos\left(\alpha_{i}\right)=\eta_{\alpha }$ as an instance. The above equation can be formulated as follows:

(5) $$\begin{array}{l} x-x_{i}-r_{i}cos\left(\alpha_{i}\right)\\ =x-x_{i}-r_{i}\;cos\left(\alpha_{i}^{0}+\varepsilon_{i}\right)\\ \approx x-x_{i}-r_{i}cos\left(\alpha_{i}^{0}\right)+r_{i}sin\left(\alpha_{i}^{0}\right)\varepsilon_{i} \end{array}$$

According to (1) and (5), we have

(6) $$\eta_{\alpha }=r_{i}sin\left(\alpha_{i}^{0}\right)\varepsilon_{i}$$

The derivation of $\eta_{\beta }$ is the same. Thus, we have

(7) $$\eta_{\beta }=r_{i}sin\left(\beta_{i}^{0}\right)\varepsilon_{i}$$

From (3)–(7), the pseudo-linear equations can be arranged as follows:

(8) $$h_{1}-G_{1}\theta =B_{1}\eta$$

where

$$h_{1}={\left[\begin{array}{ccc} x_{1} & \cdots & \begin{array}{ccc} x_{M} & y_{1} & \begin{array}{cc} \ldots & y_{M} \end{array} \end{array} \end{array}\right]}^{T},\;\;G_{1}=\left[\begin{array}{cc} G_{11} & G_{12}\\ G_{21} & G_{22} \end{array}\right],\;G_{11}={\underset{M}{\underbrace{\left[\begin{array}{ccc} 1 & \cdots & 1\\ 0 & \cdots & 0\\ 0 & \cdots & 0 \end{array}\right]}}}^{T},\;G_{21}={\underset{M}{\underbrace{\left[\begin{array}{ccc} 0 & \cdots & 0\\ 1 & \cdots & 1\\ 0 & \cdots & 0 \end{array}\right]}}}^{T},\;\theta ={\left[\begin{array}{ccc} {u_{o}}^{T} & r_{1} & \begin{array}{cc} \cdots & r_{M} \end{array} \end{array}\right]}^{T},\;\;B_{1}=\left[\begin{array}{cc} B_{11} & 0_{M\times M}\\ 0_{M\times M} & B_{12} \end{array}\right],\;\eta ={\left[\begin{array}{cc} \varepsilon^{T} & \delta^{T} \end{array}\right]}^{T}\;\;G_{12}=diag\left(\left[-\begin{array}{ccc} cos\left(\alpha_{1}\right) & \cdots & -cos\left(\alpha_{M}\right) \end{array}\right]\right),\;\;G_{22}=diag\left(\left[-\begin{array}{ccc} cos\left(\beta_{1}\right) & \cdots & -cos\left(\beta_{M}\right) \end{array}\right]\right),\;\;B_{11}=diag\left(\left[-\begin{array}{ccc} r_{1}sin\left(\alpha_{1}^{0}\right) & \cdots & -r_{M}sin\left(\alpha_{M}^{0}\right) \end{array}\right]\right),\;\;B_{12}=diag\left(\left[-\begin{array}{ccc} r_{1}sin\left(\beta_{1}^{0}\right) & \cdots & -r_{M}sin\left(\beta_{M}^{0}\right) \end{array}\right]\right),$$

We obtain the optimal θ by

(9) $$min{\left(h_{1}-G_{1}\theta \right)}^{T}W\left(h_{1}-G_{1}\theta \right)$$

where $W={\left(B_{1}QB_{1}^{T}\right)}^{-1}$ is the weighted matrix;$\;Q=E\left(\eta \eta^{T}\right)$ is the noise matrix.

Ignoring the constraint results in (9), it yields

(10) $$\theta ={\left(G_{1}^{T}WG_{1}\right)}^{-1}G_{1}^{T}Wh_{1}$$

The above optimization problem is based on the assumption that the elements in θ are independent. Nonetheless, this assumption of independence does not hold. Given the interrelation between the source position and the fundamental sensor parameters, Equation (9) is reconstituted into a constrained weighted least squares optimization problem.

(11) $$min{\left(h_{1}-G_{1}\theta \right)}^{T}W\left(h_{1}-G_{1}\theta \right)\;s.t.\left\|\theta \left(1:3\right)-s_{i}\right\|=\theta \left(i+3\right)\left(i=1,2,3,\ldots M\right)$$

The conventional two-step weighted least squares approach proves inadequate for addressing the optimization issue at hand. The complexity of the second phase escalates significantly with the increment in the estimated parameters $r_{i}$. The columns associated with the estimator z are uniformly zero. Thus, the estimate of z depends mainly on the constraint term in (11).

The objective function in (11) can be rewritten as follows:

(12) $${\left(h_{1}-G_{1}\theta \right)}^{T}W\left(h_{1}-G_{1}\theta \right)=tr\left\{\left[\begin{array}{cc} Y & \theta \\ \theta^{T} & 1 \end{array}\right]F\right\}$$

where $Y=\theta \theta^{T}\;and\;F=\left[\begin{array}{cc} G_{1}^{T}WG_{1} & {-G}_{1}^{T}Wh_{1}\\ {-h}_{1}^{T}WG_{1} & h_{1}^{T}Wh_{1} \end{array}\right]$.

Thus, the constraints in (11) can be expressed as

(13) $$Y\left(i+3,i+3\right)=tr\left(Y\left(1:3,1:3\right)\right)-2s_{i}^{T}\theta {\left(1:3\right)}^{T}+s_{i}^{T}s_{i}$$

According to the analysis in [17], the non-convex constraint $Y=\theta \theta^{T}$ can be relaxed as $Y\underline{≻}\;\theta \theta^{T}$. According to the Schur complement theorem, $Y\underline{≻}\;\theta \theta^{T}$ is equivalent to the following:

(14) $$\left[\begin{array}{cc} Y & \theta \\ \theta^{T} & 1 \end{array}\right]\underline{≻}\;0$$

To improve the tightness of the relaxed SDP problem, second-order cone constraints (SOCPs) can be added to the optimization problem. It can be represented as

(15) $$\|\theta \left(1:3\right)-s_{i}\|\leq \theta \left(i+3\right)\left(i=1,2,3,\ldots M\right)$$

After applying SDP, the optimization problem can be reformulated as follows:

(16) $$mintr\left\{\left[\begin{array}{cc} Y & \theta \\ \theta^{T} & 1 \end{array}\right]F\right\}\;s.t.\;\left(13\right),\left(14\right),\left(15\right)\left(i=1,2,3,\ldots M\right)$$

The optimal θ can be obtained through the CVX toolbox.

Through the above analysis, we can obtain the source position in the case of an unknown slant distance. To improve the estimation accuracy of the source position, a bias compensation method is also implemented, where the solution of (16) is used as an initial guess.

### 3.2. Bias Compensation Method Based on Taylor Expansion

Take the Taylor series expansion of $r_{i}=\|u_{o}-s_{i}\|$ around ${\tilde{u}}_{o}$ and retain up to the first-order terms to arrive at

(17) $$r_{i}=\|u_{o}-s_{1}\|=\|{\tilde{u}}_{0}-s_{i}\|-\rho_{{\tilde{u}}_{0},s_{i}}^{T}∆u$$

where $\rho_{{\tilde{u}}_{0},s_{i}}^{T}=\frac{{\left({\tilde{u}}_{0}-s_{i}\right)}^{T}}{\left\|{\tilde{u}}_{0}-s_{i}\right\|}$ and ${\tilde{\;u}}_{o}$ is obtained by the SDP method.

Substituting (17) and ${\tilde{u}}_{o}\;\left({\tilde{u}}_{o}=u_{o}+∆u\right)$ into (3) yields

(18) $$\left\{\begin{array}{c} \tilde{x}-∆x-x_{i}-\left(\|{\tilde{u}}_{0}-s_{i}\|-\rho_{{\tilde{u}}_{0},s_{i}}^{T}∆u\right)cos\left(\alpha_{i}\right)=\eta_{\alpha }\\ \tilde{y}-∆y-y_{i}-\left(\|{\tilde{u}}_{0}-s_{i}\|-\rho_{{\tilde{u}}_{0},s_{i}}^{T}∆u\right)cos\left(\beta_{i}\right)=\eta_{\beta } \end{array}\right.$$

The linear equations can be expressed as follows:

(19) $$h_{2}-G_{2}∆u=B_{1}\eta$$

where

$$h_{2}={\left[\begin{array}{cc} h_{21}^{T} & h_{22}^{T} \end{array}\right]}^{T},G_{2}={\left[\begin{array}{cc} G_{21}^{T} & G_{22}^{T} \end{array}\right]}^{T},\;h_{21}=\left[\begin{array}{c} \tilde{x}-x_{1}-\left\|{\tilde{u}}_{0}-s_{1}\right\|cos\left(\alpha_{1}\right)\\ \ldots \\ \tilde{x}-x_{M}-\left\|{\tilde{u}}_{0}-s_{M}\right\|cos\left(\alpha_{M}\right) \end{array}\right],\;h_{22}=\left[\begin{array}{c} \tilde{y}-y_{1}-\left\|{\tilde{u}}_{0}-s_{1}\right\|cos\left(\beta_{1}\right)\\ \ldots \\ \tilde{y}-y_{M}-\left\|{\tilde{u}}_{0}-s_{M}\right\|cos\left(\beta_{M}\right) \end{array}\right],\;G_{21}=\left[\begin{array}{c} \begin{array}{ccc} 1-\rho_{x}cos\left(\alpha_{1}\right) & -\rho_{y}cos\left(\alpha_{1}\right) & -\rho_{z}cos\left(\alpha_{1}\right) \end{array}\\ \ldots \\ \begin{array}{ccc} 1-\rho_{x}cos\left(\alpha_{M}\right) & -\rho_{y}cos\left(\alpha_{M}\right) & -\rho_{z}cos\left(\alpha_{M}\right) \end{array} \end{array}\right],\;G_{22}=\left[\begin{array}{c} \begin{array}{ccc} -\rho_{x}cos\left(\beta_{1}\right) & 1-\rho_{y}cos\left(\beta_{1}\right) & -\rho_{z}cos\left(\beta_{1}\right) \end{array}\\ \ldots \\ \begin{array}{ccc} -\rho_{x}cos\left(\beta_{M}\right) & 1-\rho_{y}cos\left(\beta_{M}\right) & -\rho_{z}cos\left(\beta_{M}\right) \end{array} \end{array}\right],\;\rho_{{\tilde{u}}_{0},s_{1}}^{T}=\left[\begin{array}{ccc} \rho_{x} & \rho_{y} & \rho_{z} \end{array}\right].$$

Because the measurement of the coefficient matrix $h_{2}$ and $G_{2}$ has noise, the solution of this method will be affected. Rearrange the coefficient matrix as follows:

(20) $$A=\left[\begin{array}{cc} -G_{2} & h_{2} \end{array}\right]\;V={\left[\begin{array}{cc} ∆u^{T} & 1 \end{array}\right]}^{T}$$

The cost function can be designed as follows:

(21) $$J=min{\left(AV\right)}^{T}WAV$$

The matrix A includes the no-noise term and the measurement noise.

(22) $$A=∆A+A^{0}$$

where $A^{0}$ is a true measurement matrix, while $∆A=\left[\begin{array}{cc} -∆G & ∆h \end{array}\right]$ represents the noise term.

(23) $$∆A=\left[\begin{array}{ccc} C_{1}\eta & C_{2}\eta & \begin{array}{cc} C_{3}\eta & C_{4}\eta \end{array} \end{array}\right]$$

where

$$C_{1}=\left[\begin{array}{cc} C_{11} & 0_{M\times M}\\ 0_{M\times M} & C_{12} \end{array}\right],C_{2}=\left[\begin{array}{cc} C_{21} & 0_{M\times M}\\ 0_{M\times M} & C_{22} \end{array}\right],\;C_{3}=\left[\begin{array}{cc} C_{31} & 0_{M\times M}\\ 0_{M\times M} & C_{32} \end{array}\right],C_{4}=\left[\begin{array}{cc} C_{41} & 0_{M\times M}\\ 0_{M\times M} & C_{42} \end{array}\right],\;C_{11}=diag\left(\left[-\begin{array}{ccc} sin\left(\alpha_{1}\right)\rho_{x} & \cdots & -sin\left(\alpha_{M}\right) \end{array}\rho_{x}\right]\right),\;C_{12}=diag\left(\left[-\begin{array}{ccc} sin\left(\beta_{1}\right)\rho_{x} & \cdots & -sin\left(\beta_{M}\right) \end{array}\rho_{x}\right]\right),\;C_{21}=diag\left(\left[-\begin{array}{ccc} sin\left(\alpha_{1}\right)\rho_{y} & \cdots & -sin\left(\alpha_{M}\right) \end{array}\rho_{y}\right]\right),\;C_{22}=diag\left(\left[-\begin{array}{ccc} sin\left(\beta_{1}\right)\rho_{y} & \cdots & -sin\left(\beta_{M}\right) \end{array}\rho_{y}\right]\right),\;C_{31}=diag\left(\left[-\begin{array}{ccc} sin\left(\alpha_{1}\right)\rho_{z} & \cdots & -sin\left(\alpha_{M}\right) \end{array}\rho_{z}\right]\right),\;C_{32}=diag\left(\left[-\begin{array}{ccc} sin\left(\beta_{1}\right)\rho_{z} & \cdots & -sin\left(\beta_{M}\right) \end{array}\rho_{z}\right]\right),\;C_{41}=diag\left(\left[\begin{array}{ccc} sin\left(\alpha_{1}\right)r_{1} & \cdots & sin\left(\alpha_{M}\right) \end{array}r_{M}\right]\right),\;C_{42}=diag\left(\left[\begin{array}{ccc} sin\left(\beta_{1}\right)r_{1} & \cdots & sin\left(\beta_{M}\right) \end{array}r_{M}\right]\right),$$

Substituting (23) into (21) yields the cost function:

(24) $$J=V^{T}{A^{0}}^{T}WA^{0}V+V^{T}{∆A}^{T}W∆AV+2V^{T}{∆A}^{T}WA^{0}V$$

The third term in *J* will vanish when we take the expectation of it. Then, the second term can be treated as a constant constraint. Thus, a new cost function can be obtained:

(25) $$minV^{T}A^{T}WAV\;s.t.\;V^{T}\Sigma V=k$$

where $\Sigma =E\left[{∆A}^{T}W∆A\right]$. *k* is a constant value.

(26) $$\Sigma =E\left[{∆A}^{T}W∆A\right]=\left[\begin{array}{cccc} \Sigma_{11} & \Sigma_{12} & \Sigma_{13} & \Sigma_{14}\\ \Sigma_{21} & \Sigma_{22} & \Sigma_{23} & \Sigma_{24}\\ \Sigma_{31} & \Sigma_{32} & \Sigma_{33} & \Sigma_{34}\\ \Sigma_{41} & \Sigma_{42} & \Sigma_{43} & \Sigma_{44} \end{array}\right]$$

where

$$\Sigma_{11}=tr\left(C_{1}WC_{1}^{T}Q\right),\Sigma_{12}=tr\left(C_{1}WC_{2}^{T}Q\right),\;\Sigma_{13}=tr\left(C_{1}WC_{3}^{T}Q\right),\Sigma_{14}=tr\left(C_{1}WC_{4}^{T}Q\right),\Sigma_{21}=\Sigma_{12}^{T},\;\Sigma_{22}=tr\left(C_{2}WC_{2}^{T}Q\right),\Sigma_{23}=tr\left(C_{2}WC_{3}^{T}Q\right),\;\Sigma_{24}=tr\left(C_{2}WC_{4}^{T}Q\right),\Sigma_{31}=\Omega_{13}^{T},\Sigma_{32}=\Sigma_{23}^{T},\;\Sigma_{33}=tr\left(C_{3}WC_{3}^{T}Q\right),\Sigma_{34}=tr\left(C_{3}WC_{4}^{T}Q\right),\Sigma_{41}=\Sigma_{14}^{T},\;\Sigma_{44}=tr\left(C_{4}WC_{4}^{T}Q\right),\Sigma_{42}=\Sigma_{24}^{T},\Sigma_{43}=\Sigma_{34}^{T}.$$

We introduce Lagrange multiplier technology to (25) and obtained the following:

(27) $$minV^{T}A^{T}WAV-\lambda \left(k-V^{T}\Omega V\right)$$

Taking the derivative concerning V and setting it to zero yields

(28) $$\left(A^{T}WA\right)V=\lambda \Omega V$$

The estimated ∆u can be obtained by generalized singular value decomposition (GSVD) theory [21].

Thus, the improved result can be obtained:

(29) $$u_{o}={\tilde{u}}_{0}-∆u$$

According to the above analysis, the whole algorithm can be summarized as Algorithm 1 shows.
**Algorithm 1:** The AOA-based localization method based on bias compensationStep 1: Set $B_{1}$ as an identity matrix and solve the optimization problem using (10). A rough value θ can be obtained.Step 2: Reformulate the weighting matrix W with the rough value θ.
Step 3: Construct constraints based on the (13)–(15).Step 4: Get the closed-form ${\tilde{u}}_{0}$ of the localization problem using (16) based on the SDP method.Step 5: Based on the closed-form solution, construct a constrained optimization equation based on the Taylor expansion as (25) shows.Step 6: Use Lagrange multiplier technology to get the bias ∆u as (27) shows.Step 7: Revise the result using (29) and an improved result can be obtained.

### 3.3. CRLB Analysis

The CRLB is given by

(30) $$\mathrm{CRLB}\left(u_{0}\right)=FIM^{-1}\left(u_{0}\right)$$

where FIM can be represented as

(31) $$FIM\left(u_{0}\right)={\left(\frac{\partial m^{o}}{\partial u_{0}}\right)}^{T}Q^{-1}\left(\frac{\partial m^{o}}{\partial u_{0}}\right)$$

where $m^{o}=\left[\alpha^{o};\beta^{o}\right]$ is the real bearing without any noise.

The detailed derivation of $\frac{\partial m^{o}}{\partial u_{0}}$ can be found in Appendix A.

## 4. Simulation and Field Test

### 4.1. Simulation Test

In this section, a series of simulation experiments are designed to verify the effectiveness and improvement of the proposed method. The CRLB is plotted as a benchmark.

The symbols used in the simulation experiment are listed as follows:

‘WLS’ represents the weighted least-squares method, as (10) shows, which is used in paper [5].

‘SDP’ denotes the semidefinite programming method described in Section 3, which is used in paper [19].

‘proposed’ denotes the bias compensation method based on the SDP result.

We use the root mean square error (RMSE) and the bias norm of the source position as the performance metric.

(32) $$\mathrm{RMSE}\left({\hat{u}}_{o}\right)=\sqrt{\frac{\sum_{i=1}^{L}\|{\hat{u}}_{o}-{u_{o}}^{2}\|}{L}}\;\mathrm{BiasNorm}=\|\;\frac{\sum_{i=1}^{L}{\hat{u}}_{o}}{L}-u_{o}\|$$

where *L* denotes the number of tests (*L* = 200). $u_{o}$ is the true source position.

The covariance matrix of the bearing noise is modeled as

(33) $$Q=diag\left(\begin{array}{cc} \sigma_{s}^{2}I_{M} & \sigma_{s}^{2}I_{M} \end{array}\right)$$

The sensor position model is as follows:

(34) $$s_{i}={\left[\begin{array}{cc} 100cos\left(\frac{\pi }{4}\left(i-2\right)\right) & \begin{array}{cc} 100sin\left(\frac{\pi }{4}\left(i-2\right)\right) & 50z_{i} \end{array} \end{array}\right]}^{T}$$

where i denotes the sensor number.$\;z_{i}\in \left(-1,1\right)$ denotes a set of random numbers. 

The reference sensor $s_{1}$ is located at ${\left[\begin{array}{cc} 0 & \begin{array}{cc} 0 & 0 \end{array} \end{array}\right]}^{T}$. The source position is chosen randomly as ${\left[\begin{array}{cc} 150 & \begin{array}{cc} 200 & 10 \end{array} \end{array}\right]}^{T}$.

#### 4.1.1. Simulation 1—Analysis under Different AOA Noises

The positioning accuracy is affected by noise. In this test, sensor number M = 6. We compared the RMSE of the estimated source position with different methods.

**Figure 3:**
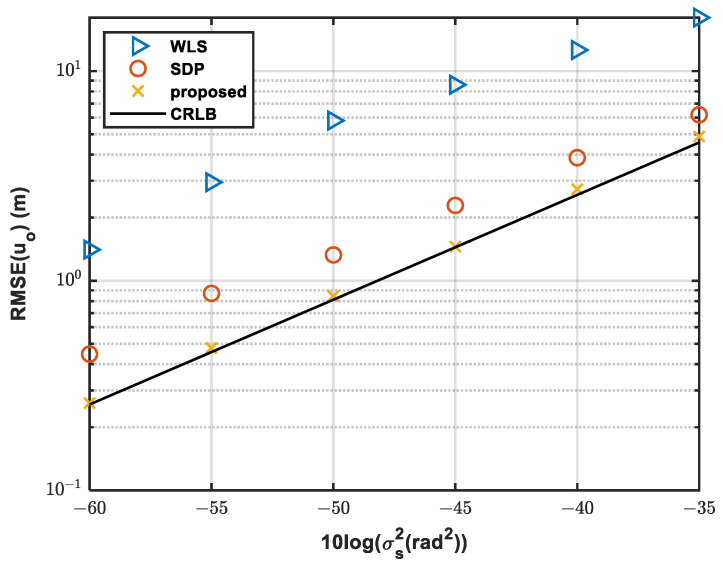
Comparison of RMSE with different sensor noises.

Figure 3 shows the comparison of the RMSEs of different methods. The positioning accuracy deteriorates with the increase in noise. The proposed method can achieve equal performance with the CRLB no matter how the noise changes. However, the SDP method and WLS method have a certain bias from the CRLB. Given that the SDP method incorporates parameter constraint relations, it achieves superior positioning accuracy compared to the WLS method. Consequently, the SDP outcomes serve as more appropriate initial values for bias reduction techniques. The proposed method shows a superior performance, e.g., when $10\log\left(\sigma_{s}^{2}\right)\;$= −35, the RMSE of the proposed method is 4.88 m, while that using SDP is 6.18 and using WLS is 18.01 m. Compared with the SDP method and the WLS method, the root mean square error decreased by 1.3 m and 13.13 m. 

**Figure 4:**
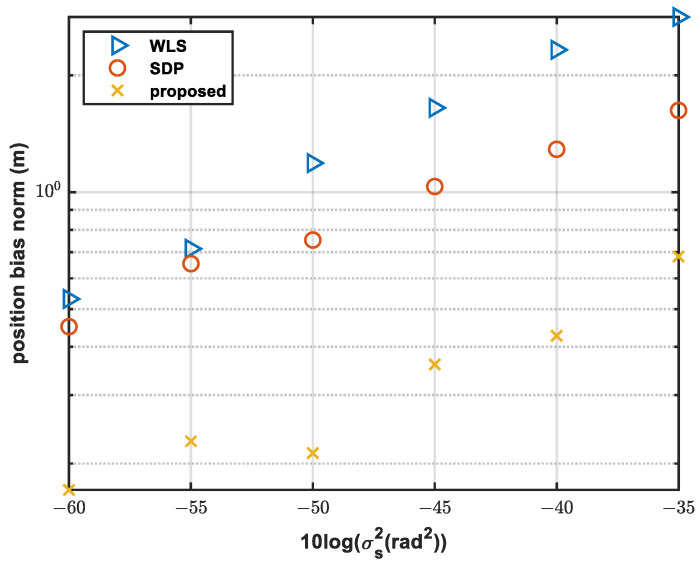
Comparison of bias norm with different sensor noises.

Figure 4 shows the bias norm of the source position.

It can be seen from Figure 4 that the positioning bias increases with the increase in noise. The original solution suffers from the noisy measurement and has a large bias in the estimated results. However, the proposed method can effectively suppress the positioning bias caused by noise. It is obvious that the proposed method has the smallest bias, e.g., when $10\log\left(\sigma_{s}^{2}\right)$ = −35, the bias norm of the proposed method is 0.68 m, while that with SDP is 1.62 and with WLS is 2.83 m. Compared with the SDP method and the WLS method, the bias norm is decreased by 0.94 m and 2.15 m.

The advantage of the SDP method is that it can estimate the source position without the range information. Although the solution of the SDP method cannot attain the CRLB, it can serve as the initialization for the bias compensation method and the proposed method can improve the estimation accuracy.

#### 4.1.2. Simulation 2—Analysis under Different Sensor Numbers 

In this sub-section, $\sigma_{s}^{2}\;$is set as an invariable value $\left(\sigma_{s}^{2}=0.0001rad^{2}\right)$, while the number of sensors is changed from 8 to 12.

We recorded the RMSE of the source position estimated by the different methods. 

**Figure 5:**
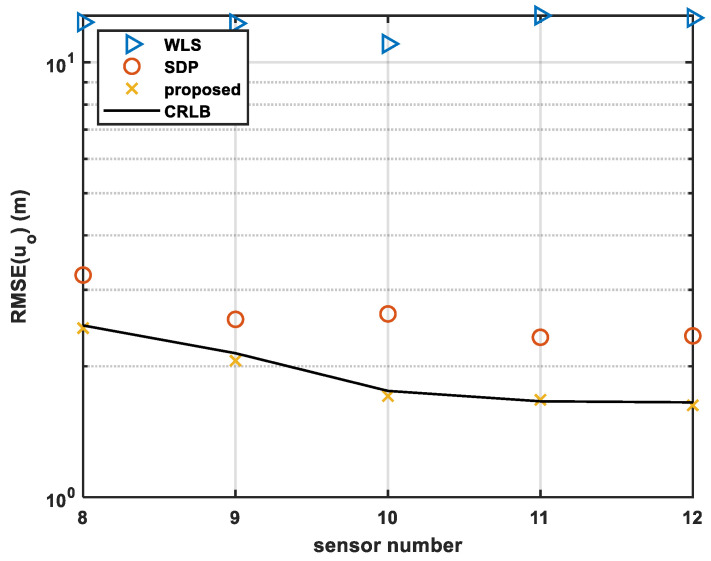
Comparison of RMSE with different sensor numbers.

As can be observed from Figure 5, the proposed method achieves the same performance as the CRLB regardless of the number of sensors. However, the traditional methods have certain biases from the CRLB. As the constraint relation of the parameter is not considered in the WLS method, it has a larger positioning error than the other methods. It can be observed that in the case of 12 sensors, the RMSE of the proposed method is 1.63 m, while that using SDP is 2.35 m and with WLS is 12.66 m. Additionally, according to the curve trend in the figure, it can be concluded that with the increase in the number of sensors, the error convergence of the algorithm has the best performance. From the analysis of the improvement in positioning accuracy, compared with the SDP algorithm and WLS algorithm, the algorithm proposed in this paper improves by 30.6% and 87.1%, respectively.

**Figure 6:**
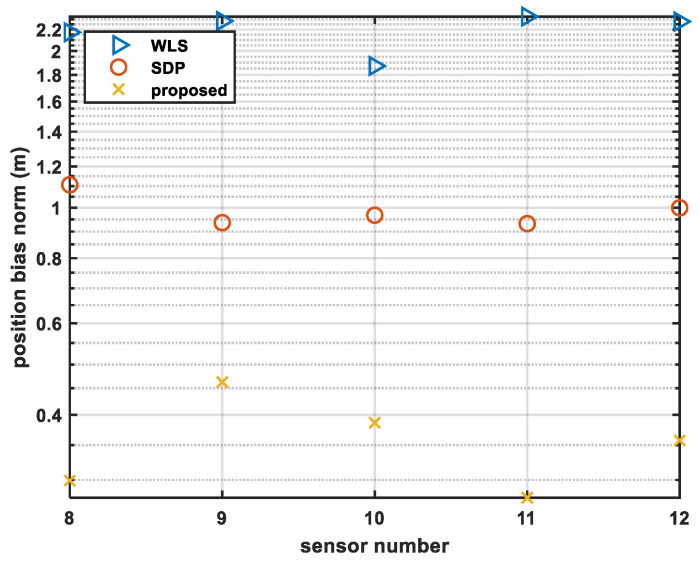
Comparison of bias norm with different sensor numbers.

We recorded the bias norm of the source position estimated by the different methods, as Figure 6 shows.

It can be seen from Figure 6 that the original solution suffers from the noisy measurement and has a large bias in the estimated results. However, the proposed method can effectively suppress the positioning bias caused by noise. It is obvious that the proposed method has the smallest bias, e.g., when the sensor number is 12, the bias norm of the proposed method is 0.36 m, while that using SDP is 1.00 and with WLS is 2.28 m. Compared with the SDP method and the WLS method, the bias norm is decreased by 0.64 m and 1.92 m.

The results of test 1 and test 2 confirm that the proposed novel scheme can locate the source position when the range measurement is unavailable. Additionally, the solution can achieve CRLB accuracy well.

#### 4.1.3. Simulation 3—Analysis of the Algorithms’ Computational Efficiency

Generally, the shorter the running time of the algorithm, the higher the computational efficiency. Thus, a comparison of the computation time of the algorithms on the Matlab platform was conducted. The computational efficiency was compared by the computation time. The running times of the algorithms in the condition of Simulation 1 are compared in Table 1.

Table 1 compares the time cost on the Matlab R2023b platform. It shows that the computational cost of WLS is less than that of the proposed method. This is because the proposed method uses the initial value obtained from the SDP method. The SDP method is more accurate, but requires more computation time. In engineering, if it is configured with some high-performance computing chips, which will meet the requirements of the computational efficiency, the proposed method is an alternative.

### 4.2. Field Test

In this section, a field test is designed to verify the effectiveness and improvement of the proposed method. The field test was carried out in the Yangtze River.

As described in References [3,22], the USBL system can provide two bearing angles and it can be applied to the underwater localization problem. Considering the positioning problem, which relies solely on angle-of-arrival (AOA) measurements when slant distance information is absent, subsequent experiments will focus exclusively on the bearing measurements provided by the USBL system.

**Figure 7:**
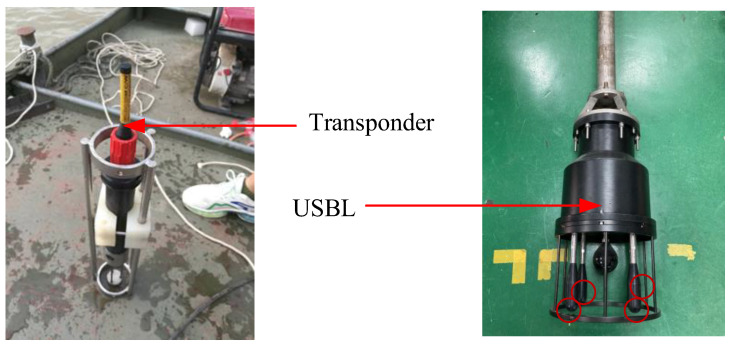
Diagram of the USBL equipment.

The USBL equipment is shown in Figure 7.

The error parameters of the USBL system are as follows:

USBL:

Positioning error: 0.1 m + 1%r;

Bearing error: 0.2°.

**Figure 8:**
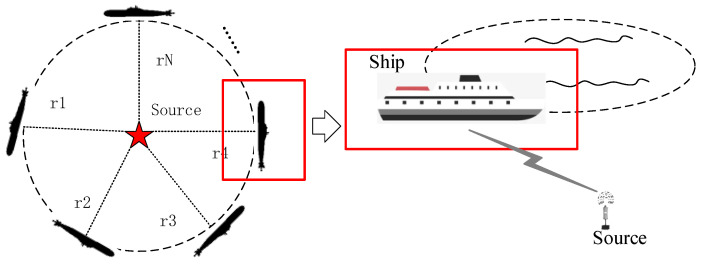
Schematic diagram of the underwater source localization problem.

The four transducers are distributed along the x-axis and y-axis, which are marked with a red circle. The distribution of the transducer array is the same as that in Figure 2. The transponder is placed on the bottom of the river and is treated as a source with an unknown position. As shown in Figure 8, a ship equipped with high-precision RTK voyages around the source. When the ship moves on the water surface, each time the USBL measurement data are received, the GPS position and attitude value are recorded at this time. After a while, several ship positions along the trajectory are recorded and they can form an array to be used for the calculation of the source position. Thus, the AOA-based localization problem can be treated as an underwater source localization problem with the known ship position. The goal is to locate the transponder position with the bearing angles and the ship position.

The true transponder position was calibrated in advance and can be used to evaluate the accuracy of AOA positioning. The detailed calibration process can be found in [23]. The calibration accuracy can reach the level of a centimeter; it can be used as the truth value of algorithm verification.

**Figure 9:**
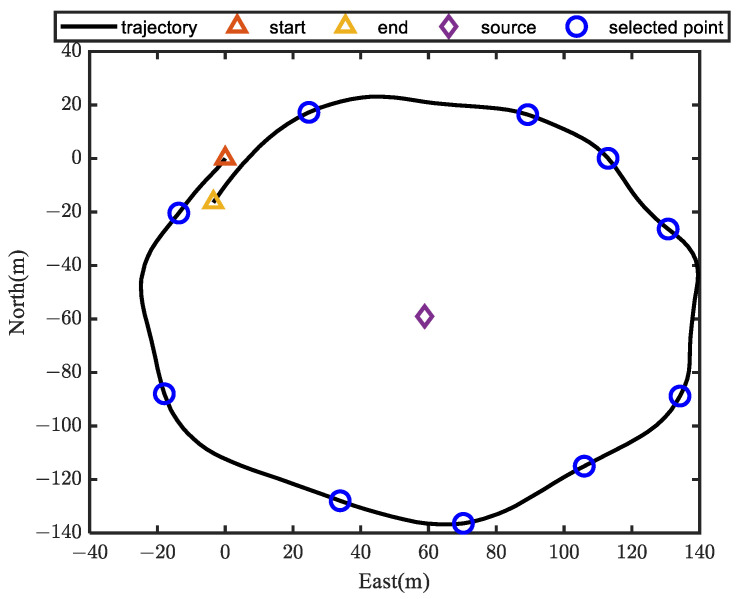
Schematic diagram of localization scenario.

The ship’s trajectory in rectangular coordinates is shown in Figure 9, and is marked as a black line. Several ship positions along the trajectory are selected as the reference, and are marked as blue circles. The selected ship position and the transponder position are also shown in Figure 9. In this experiment, nine ship positions are selected to calculate the source position. Although different numbers of positions will affect the positioning accuracy, it is convincing that all the algorithms are evaluated under the same numbers. Thus, in the experiment, the AOA-based localization problem can be treated as a localization problem using the nine reference positions with the bearing measurements.

**Figure 10:**
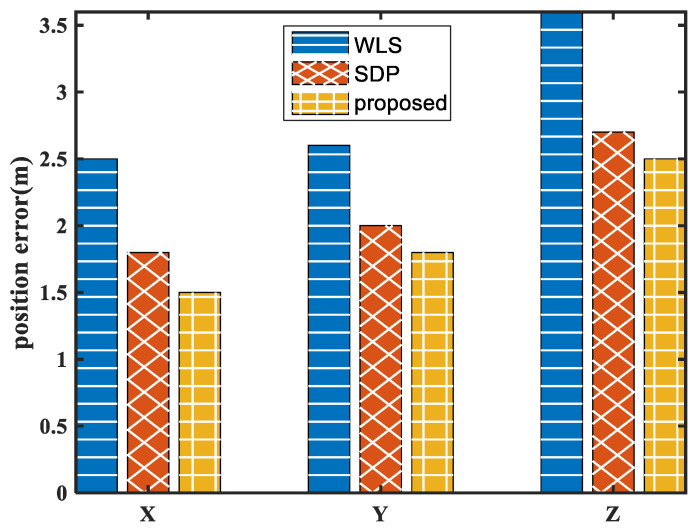
Position error of the field experiment.

The true source position is calibrated in advance and it can be used as the truth value of algorithm verification. Thus, the errors of the estimated position and the true position of different algorithms are used as the performance metrics used to evaluate the proposed method. The triaxial position error with different algorithms is shown in Figure 10.

It can be seen from Figure 10 that the proposed method obtains the highest positioning accuracy. Compared with the WLS method, the position accuracy is improved by 40%, 30%, and 30% in the X, Y, and Z directions, respectively. Compared with the SDP method, the position accuracy is also improved by 17%, 10%, and 7% in the X, Y, and Z directions, respectively.

A simple semi-physical simulation experiment verified the performance of the algorithms in the underwater environment. It proves that the proposed method in this paper can locate the source position independently of the slant distance. Compared with the traditional positioning method based on bearing and slant distance, the application scope is broadened.

## 5. Conclusions

The source localization problem in 3-D space is addressed in this paper. Contrary to conventional AOA localization models that rely on azimuth and elevation measurements, this study analyzes a novel positioning model utilizing solely bearing measurements. The challenge of AOA positioning using the bearings is that it needs slant distance information to complete 3-D positioning. The proposed method enables Ultra-Short Baseline (USBL) positioning solely through bearing measurements, even in the absence of slant distance information. This method exhibits superior positioning accuracy compared to traditional approaches. The CRLB is used as the benchmark of the comparison algorithm in this paper, and the simulation results show that the proposed method can achieve better performance than the traditional method. Field tests further validate that the proposed method can accurately locate the source position without range measurements, achieving the highest level of positioning accuracy.

In the future, the proposed method can be applied in underwater acoustic positioning, especially in the USBL positioning system without slant distance information. It can provide high precision attitude and position information for underwater vehicles. It can also be applied in passive navigation or the acoustical localization of the black box.

## Figures and Tables

**Table 1:** Comparison of the computation time.

Algorithm	WLS (s)	SDP (s)	Proposed (s)
Running Time	0.02	3.97	3.99

## Data Availability

Data are contained within the article.

## Appendix A

The detailed derivation of $\frac{\partial m^{o}}{\partial u_{0}}$ is as follows:

(A1) $$\frac{\partial m^{o}}{\partial u_{0}}=\left[\begin{array}{c} \frac{\partial \alpha^{o}}{\partial u_{0}}\\ \frac{\partial \beta^{o}}{\partial u_{0}} \end{array}\right]={\left[\begin{array}{ccc} {\left(\frac{\partial \alpha_{1}^{o}}{\partial u_{0}}\right)}^{T} & \ldots & \begin{array}{ccc} {\left(\frac{\partial \alpha_{M}^{o}}{\partial u_{0}}\right)}^{T} & {\left(\frac{\partial \beta_{1}^{o}}{\partial u_{0}}\right)}^{T} & \begin{array}{cc} \cdots & {\left(\frac{\partial \beta_{M}^{o}}{\partial u_{0}}\right)}^{T} \end{array} \end{array} \end{array}\right]}^{T}$$

Take $\frac{\partial \alpha_{i}^{o}}{\partial u_{0}}$ as an example. $\left(i=1,2,\ldots M\right)$

(A2) $$\frac{\partial \alpha_{i}^{o}}{\partial u_{0}}=\left[\begin{array}{ccc} \frac{\partial \alpha_{i}^{o}}{\partial x} & \frac{\partial \alpha_{i}^{o}}{\partial y} & \frac{\partial \alpha_{i}^{o}}{\partial z} \end{array}\right]$$

where

$$\frac{\partial \alpha_{i}^{o}}{\partial x}=a\left(\frac{1}{\left\|u_{0}-s_{i}\right\|}-\frac{{\left(x-x_{i}\right)}^{2}}{{\left({\left(u_{0}-s_{i}\right)}^{T}\left(u_{0}-s_{i}\right)\right)}^{1.5}}\right).\;\frac{\partial \alpha_{i}^{o}}{\partial y}=a\left(-\frac{\left(x-x_{i}\right)\left(y-y_{i}\right)}{{\left({\left(u_{0}-s_{i}\right)}^{T}\left(u_{0}-s_{i}\right)\right)}^{1.5}}\right).\;\frac{\partial \alpha_{i}^{o}}{\partial z}=a\left(-\frac{\left(x-x_{i}\right)\left(z-z_{i}\right)}{{\left({\left(u_{0}-s_{i}\right)}^{T}\left(u_{0}-s_{i}\right)\right)}^{1.5}}\right).\;a=\frac{-1}{\sqrt{1-{\left(\frac{x-x_{i}}{\left\|u_{0}-s_{i}\right\|}\right)}^{2}}}.$$

Due to the fact that $\frac{\partial \beta_{i}^{o}}{\partial u_{0}}$ has the same form with $\frac{\partial \alpha_{i}^{o}}{\partial u_{0}}$, $\frac{\partial \beta_{i}^{o}}{\partial u_{0}}$ can be expressed as

(A3) $$\frac{\partial \beta_{i}^{o}}{\partial u_{0}}=\left[\begin{array}{ccc} \frac{\partial \beta_{i}^{o}}{\partial x} & \frac{\partial \beta_{i}^{o}}{\partial y} & \frac{\partial \beta_{i}^{o}}{\partial z} \end{array}\right]$$

where

$$\frac{\partial \beta_{i}^{o}}{\partial x}=b\left(-\frac{\left(x-x_{i}\right)\left(y-y_{i}\right)}{{\left({\left(u_{0}-s_{i}\right)}^{T}\left(u_{0}-s_{i}\right)\right)}^{1.5}}\right).\;\frac{\partial \alpha_{i}^{o}}{\partial y}=b\left(\frac{1}{\left\|u_{0}-s_{i}\right\|}-\frac{{\left(y-y_{i}\right)}^{2}}{{\left({\left(u_{0}-s_{i}\right)}^{T}\left(u_{0}-s_{i}\right)\right)}^{1.5}}\right).\;\frac{\partial \alpha_{i}^{o}}{\partial z}=b\left(-\frac{\left(y-y_{i}\right)\left(z-z_{i}\right)}{{\left({\left(u_{0}-s_{i}\right)}^{T}\left(u_{0}-s_{i}\right)\right)}^{1.5}}\right).\;b=\frac{-1}{\sqrt{1-{\left(\frac{y-y_{i}}{\left\|u_{0}-s_{i}\right\|}\right)}^{2}}}$$
